# Clinical manifestations, laboratory markers, and renal ultrasonographic examinations in 1-month to 12-year-old Iranian children with pyelonephritis: a six-year cross-sectional retrospective study

**DOI:** 10.1186/s12879-021-05887-1

**Published:** 2021-02-18

**Authors:** Daryoosh Fahimi, Leila Khedmat, Azadeh Afshin, Zahra Noparast, Maryam Jafaripor, Effat Hosseinali Beigi, Maryam Ghodsi, Anahita Izadi, Sayed Yousef Mojtahedi

**Affiliations:** 1 Children’s Hospital Medical Centre, Tehran University of Medical Sciences, Tehran, Iran; 2 Health Management Research Center, Baqiyatallah University of Medical Sciences, Tehran, Iran; 3 Department of Pediatric Nephrology, Bahrami Hospital, Tehran University of Medical Sciences, Tehran, Iran; 4 Faculty of Medicine, Tehran University of Medical Sciences, Tehran, Iran; 5 Department of Pediatric Intensive Care Unit, Bahrami Children’s Hospital, Tehran University of Medical Sciences, Tehran, Iran; 6 Department of Pediatric Infection Disease, Tehran University of Medical Sciences, Tehran, Iran

**Keywords:** Acute pyelonephritis, Urinary tract infection, Renal pathology, Clinical signs, Ultrasound, Pediatric

## Abstract

**Background:**

Upper urinary tract infection (UTI) or pyelonephritis may increase the pathogenesis rate and risk of severe complications in children due to kidney atrophy.

**Objective:**

A set of clinical symptoms, laboratory markers, and ultrasound findings were assessed to achieve the early diagnosis and prognosis of pyelonephritis in hospitalized pediatrics.

**Methods:**

A cross-sectional study with 104 Iranian children (95 girls and 9 boys) aged 1 month to 12 years with acute pyelonephritis during 2012–2018 was conducted. The ultrasound examination of kidneys and urinary tract during hospitalization, the incidence of clinical symptoms, and laboratory markers in blood and urine were monitored to identify the best predictive factors of early diagnosis of this bacterial infection.

**Results:**

Three-fourth of the patients had one of the four clinical symptoms of abdominal pain, constipation, dysuria, and vomiting, while others were asymptomatic. A much frequency of pyuria (88.46%), *Escherichia coli* in urine (92.31%), leukocytosis (81.73%), and high ESR (> 10 mm/h, 92.30%) and CRP (> 10 mg/L, 82.82%) was observed. The kidney and urinary tract ultrasonography only in 32.7% of children revealed findings in favor of pyelonephritis (cystitis, ureteral stones, and hydronephrosis).

**Conclusion:**

There was a high frequency of clinical signs and laboratory markers associated with pyelonephritis. Ultrasound alone was not an efficient tool to track febrile UTI as most patients presented normal sonography.

## Background

Febrile urinary tract infections (UTIs) or acute pyelonephritis (AP) is one of the most common bacterial infections of the renal pelvis and kidney among children and young adult women [1]. It has been reported that the annual cost of treating this disease in France and the US was about €58 million and $2.47 billion, respectively [2, 3]. In childhood, this disease usually occurs in boy infants due to congenital anomalies of kidneys and urinary tract, while AP is observed in girls at older ages [4–6]. *Escherichia coli* is the main responsible pathogen for AP. This pathogen often causes severe inflammation and short-term (e.g., fever, dysuria, and flank pain) and long-term (e.g., irreversible renal scarring) morbidities [7, 8]. The renal scarring significantly develops the risk of hypertension, proteinuria, vesicoureteral reflux (VUR), chronic kidney disease, and end-stage renal disease [9, 10]. Since renal scarring is observed in 37% of AP children after two years from the infection onset, the rapid diagnosis and implementation of effective therapeutic measures are necessary to manage this disease among children [4, 11–13].

Although some complications such as dysuria, urgency, hesitancy, small-volume voids, or lower abdominal pain were recognized in children with UTI at older ages, infants involved with this disease usually appear non-specific symptoms like fever, failure to thrive, jaundice, irritability, and vomiting [14, 15]. On the other hand, the definitive analysis among infants with UTI involves bladder catheterization due to the nonspecific signs and symptoms of UTI in pediatric populations. Besides, clinical strategies driven by antibiotic prophylaxis or imaging tools have been recently implemented for hospitalized children with UTIs. Accordingly, several imaging techniques such as ultrasound, micturition cystourethrogram, and Tc-99 m dimercaptosuccinic acid along with a broad spectrum of intravenous prophylactic antibiotics were recommended to monitor and treat febrile UTIs (e.g., VUR and renal scarring) in children [8, 16].

Although there are many studies on the association between demographic and laboratory factors and the AP severity in children, little evidence on the combination of findings obtained from clinical manifestations, markers, and renal sonography among Iranian children within a wide age range has been published. For the first time, the efficiency of ultrasonographic examinations and antibiotics to diagnose and treat AP among Iranian infants and children was first conducted based on a high number of clinical manifestations, laboratory markers, and complications. As a result, the objective of this six-year cross-sectional study was to scrutinize the febrile UTI in 1-month to 12-year-old Iranian children based on clinical outcomes and signs, as well as renal ultrasound findings to adopt clinical practices and diagnostic tools/markers for a notable contribution to the early disease prognosis, monitoring, and treatment in the future.

## Methods

### Study design and participants

A cross-sectional study was designed to evaluate laboratory signs, clinical symptoms, and ultrasonography examinations obtained from 104 children with AP who were hospitalized in Bahrami Hospital (Tehran, Iran) from 2012 to 2018. The sampling method was the census so that all the admitted children with AP were assessed to find early prognosis and diagnosis markers. The number of collected samples was adequate since several previous studies with the same count of participants were successfully implemented [17–19]. The verbal and written informed consent using phone contacts and face-to-face interviews was obtained from all the parents after mentioning the study objective and used methodology. This research was performed following the Declaration of Helsinki and approved by the Human Ethics Committee of the Tehran University of Medical Sciences (TUMS).

### Inclusion and exclusion criteria

In this study, only pediatric patients with AP aged from 1 month to 12 years old were included. The AP was diagnosed after the specialist’s approval according to an axillary temperature of higher than 38.5 °C, bad general conditions, and positive urine culture (PUC). For the definition of PUCs, the significant microbial growth was considered based on standard microbiological criteria. Accordingly, the colony count in the midstream urine sample was more than or equal to 10^5^ CFU/mL of a single pathogen, or ≥ 10^4^ CFU/mL microorganisms counted with the reference catheter method, and/or ≥ 10^3^ Gram-positive bacteria in urine culture taken by the suprapubic method [20]. Besides, children with negative urine culture (NUC) and those who had an NUC after the antibiotic administration were excluded from the study.

### Data collection

The medical information of all patients with AP was completed from archived electronic files from March 2012 to March 2018. After ensuring the pyelonephritis diagnosis accuracy, the necessary information was extracted by referring to the medical history, disease course, and summary of patients’ files. Patients were included in the pre-prepared questionnaire form if they met the inclusion criteria. This questionnaire consisted of the patient’ name, gender, age, height, weight, body mass index (BMI, kg/m^2^), hospitalization stay, fever degree, fever duration before and after the antimicrobial therapy, drug treatment (intravenous antibiotic type), and the history of having constipation, dysuria, vomiting, and abdominal pain during hospitalization. Children were classified into four classes of underweight, normal, overweight, and obese based on the BMI reported by the Centers for Disease Control and Prevention (CDC) growth charts [16]. Also, the results of laboratory markers such as urine culture (negative/positive), microorganism type in PUC, urine analyses (e.g., pyuria (white blood cells (WBCs) per mm^3^), hematuria (red blood cells (RBCs) per high-power field (HPF)), and positive nitrite), and levels of hematological factors (e.g., hemoglobin (Hb, ng/mL), erythrocyte sedimentation rate (ESR, mm/h), and C-reactive protein (CRP, mg/L), WBCs (count per mm^3^), blood urea nitrogen (BUN, mg/dL), serum creatinine (SCr, mg/dL), potassium (K, mEq/L), and sodium (Na, mEq/L) were recorded. Then, findings obtained from ultrasound examinations of kidneys and urinary tract in terms of renal anomalies, cystitis, ureteral stones, and hydronephrosis during hospitalization stay were collected. Ultrasound imaging was conducted by two ultrasonographers. The first observer was blinded to the results of the ultrasound examination of the second observer. The inter-observer agreement between observers of differing levels of expertise was assessed according to the kappa statistic. Lastly, the kidney function level was assessed according to the estimated glomerular filtration rate (eGFR, mL/min/1.73 m^2^) using the following equation [21]:

1 $$ eGFR=\frac{0.413\times Height(cm)}{SCr\ \left( mg/ dL\right)} $$

### Statistical analysis

The one-sample Kolmogorov-Smirnov test was used to evaluate the normality of variables distribution. Data were examined at a significance level of *p* < 0.05. The descriptive data were expressed as frequency, percentage, and mean ± standard deviation. The statistical differences were determined using the independent t-test for continuous variables and Pearson’s Chi-square (χ^2^) test for categorical variables. The one-sample Kolmogorov-Smirnov test was used to evaluate the data distribution normality. The non-parametric, Mann–Whitney test was used for the data analysis with an abnormal distribution. Pearson’s coefficient was considered to find significant correlations between tested variables.

## Results

The average age of 104 patients with AP was 47.08 years. Nighty-five (91.34%) of the studied patients were girls. The minimum, mean, and maximum values of patients’ height and weight were 52 cm and 3.3 kg, 94.26 cm and 15.77 kg, and 147 cm and 69.9 kg, respectively. The highest and lowest BMI were respectively calculated to be 10 and 28 kg/m^2^, while the mean BMI of the total population with AP was 16.29 kg/m^2^ (Table 1). Table 2 exhibits the frequency of BMI classes as a function of gender differences and age groups (< 1, 1–5, and >  5 years old). Most children (girls, 62.1%; boys, 44.4%) had a normal range of BMI. Also, almost equal numbers of children were in different age ranges (31.73–34.61%, Table 2). Based on the age group classification, most overweight children had an age of less than one-year-old (*n* = 23) and over 5 years old (*n* = 23) (Table 2).

**Table 1:** The demographic and laboratory parameters of children with AP

Parameter ^a^	Frequency (n [%])	Values		
		Mean (±SD)	Minimum	Maximum
Gender-boy	9 [8.66]	–	–	–
Gender-girl	95 [91.34]	–	–	–
Age (month)	–	47.08 ± 41.49	1	144
Height (cm)	–	94.26 ± 26.36	52	147
Weight (kg)	–	15.77 ± 9.87	3.3	69.9
BMI (kg/m^2^)	–	16.29 ± 2.78	10	28
Fever (°C)	–	39.41 ± 2.51	38.5	40.9
Fever duration (BT, day)	–	4.23 ± 1.54	1	7
Fever duration (AT, day)	–	3.54 ± 0.47	0	5
HS (day)	–	5.41 ± 4.14	2	16
WBC (count per mm^3^)	–	14.90 ± 5.73	3.6	34.0
Hb (ng/mL)	–	11.02 ± 1.32	7.6	14.7
ESR (mm/h)	–	46.52 ± 26.42	3	108
CRP (mg/L)	–	58.73 ± 36.81	1	118
BUN (mg/dL)	–	11.31 ± 7.43	5	62
SCr (mg/dL)	–	0.57 ± 0.23	0.05	2.40
Na (mEq/L)		140.70 ± 3.41	131	152
K (mEq/L)		4.36 ± 0.35	3.5	5.8
eGFR (mL/min/1.73 m^2)^	–	68.94 ± 18.11	17.2	134.0

^a^*BMI* Body mass index, *HS* Hospitalization stay, *BT/AT* Before/After treatment, *WBC* White blood cell, *Hb* Hemoglobin, *ESR* Erythrocyte sedimentation rate, *CRP* C-reactive protein, *BUN* Blood urea nitrogen, *SCr* Serum creatinine, *eGFR* Estimated glomerular filtration rate

**Table 2:** The frequency of BMI classes of children with AP within different gender and age groups^a^

BMI group	Gender		Total	Age group			Total
	Girl	Boy		< 1 yr old	1-5 yrs old	> 5 yrs. old	
Underweight	13 (12.50)	2 (1.92)	15 (14.42)	2 (1.92)	5 (4.81)	8 (7.70)	15 (14.42)
Normal	59 (56.72)	4 (3.86)	63 (60.58)	23 (22.11)	17 (16.34)	23 (22.12)	63 (60.57)
Overweight	16 (15.38)	1 (0.96)	17 (16.34)	6 (5.77)	9 (8.66)	2 (1.92)	17 (16.35)
Obese	7 (6.74)	2 (1.92)	9 (8.66)	5 (4.81)	2 (1.92)	2 (1.92)	9 (8.66)
Total	95 (91.34)	9 (8.66)	104 (100)	36 (34.61)	33 (31.73)	35 (33.66)	104 (100)

^a^ Frequency was represented as: count (percentage)

**Fig. 1:**
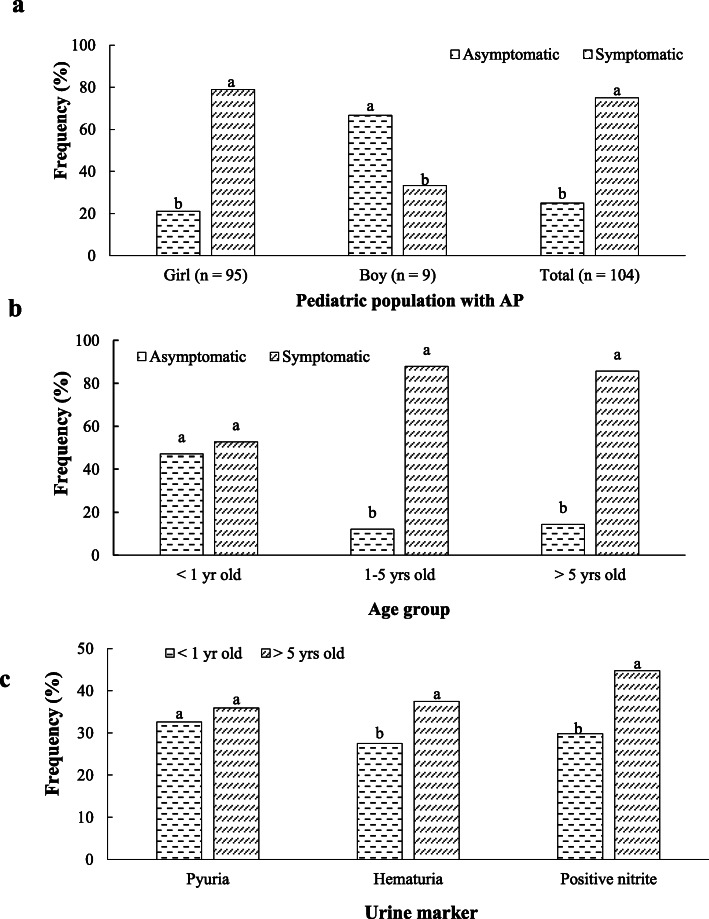
The frequency ofsymptomatology of the children population with AP based on gender (**a**) and age range (**b**), and the percentage of positive urine markers in age groups (**c**)

The minimum and maximum time of hospitalization stay were 2 days (*n* = 9) and 16 days (*n* = 1), respectively (Table 1). In general, 75% of the patients with AP were symptomatic. A high number of girls (78.9%) were symptomatic, while most boys (66.7%) were asymptomatic (Fig. 1a). This finding indicates that UTIs may occur in boys with fewer clinical symptoms. Thus, this population group needs further laboratory investigations. Also, the percentage of asymptomatic children in three age groups of < 1, 1–5, and >  5 years old was 47.2, 12.1, and 14.3%, respectively (Fig. 1b). Hence, asymptomatic was more common at younger ages. The symptomatology rate was increased with increasing age, although there was no significant difference in this index between age groups of 1–5 and > 5 years old. As 20.19% (*n* = 21) of the patients with AP complained of constipation, this symptom can be considered as a risk factor for UTIs. Fever as the main inclusion criterion for all patients averagely was at 39.4 °C, at admission time, while the minimum and maximum temperature degrees were 38.5 and 41.0 °C, respectively. The shortest and longest fever durations before the treatment were 1 day (*n* = 10) and 7 days (*n* = 6), respectively (Table 1). Although the child’s fever duration before starting the antimicrobial therapy does not play a role in confirming or rejecting a UTI, a delay in beginning the treatment can significantly have consequences like renal scarring. Three cases did not show fever after the treatment, while fever in the two other cases continued for up to 5 days after the treatment, showing a necessity to change the used antibiotic type. As dysuria was observed among 30 patients with AP (28.84%), the absence of dysuria does not rule out a UTI. Abdominal pain and vomiting were detected in 33.65% (*n* = 35) and 40.38% (*n* = 42) of the subjects, respectively (Table 3). Based on Pearson’s Chi-square analysis, there were significant associations between patients’ age and symptoms of dysuria (*p* = 0.003) and abdominal pain (*p* = 0.0001).

**Table 3:** A summary of urine markers, clinical complications, and ultrasound findings of children with AP

Findings	Observed		Non-observed	
	Frequency (n)	Percentage (%)	Frequency (n)	Percentage (%)
Urine marker				
Pyuria	92	88.46	12	11.54
Hematuria	40	38.46	64	61.54
Positive nitrite	47	45.19	57	54.81
Clinical complication				
Abdominal pain	35	33.65	69	66.35
Constipation	21	20.19	83	79.81
Dysuria	30	28.84	74	71.16
Vomiting	42	40.38	62	59.62
Ultrasound				
Hydronephrosis	22	21.15	82	78.85
Cystitis	9	8.65	95	91.35
Stone	0	0.00	104	100
Anomaly	2	1.92	102	98.08

Pyuria and hematuria usually respectively are the presence of ≥10 WBCs/mm^3^ and ≥ 5RBCs/HPF in a urine specimen. Pyuria, hematuria, and positive nitrite were respectively diagnosed in 88.46% (*n* = 92), 38.46% (*n* = 40) and 45.19% (*n* = 47) of urine tests of patients with AP (Table 3). As a result, not only pyuria can be one of the most important symptoms of UTI, but also not having hematuria and urine nitrite do not rule out this disease. Figure 1c shows that these abnormal urinary changes were increased by increasing the age from < 1 to > 5 years old. In addition, there was a negative correlation between and eGFR and patients’ age (r = 0.754, *p* = 0.001). Results proved that this increase in the urine nitrite (50.0%) was more evident than hematuria (36.36%) and pyuria (10.12%) (Fig. 1c). *E. coli* was the most frequent pathogen in urine samples so that this bacterium was present in samples of 96 patients (92.31%). Other bacteria such as Gram-negative *Bacillus* (2 cases), Group-B *Streptococcus* (2 cases), *Klebsiella pneumoniae* (1 case), *Acinetobacter* (1 case), *Enterococcus* (1 case), and *Staphylococcus aureus* (1 case) were observed in urine samples taken from patients less than one-year-old (Table 4). Ceftizoxime was the most common intravenous antibiotic to treat patients with AP (*n* = 78, 75.0%). Other used antibiotics were ceftriaxone (*n* = 12, 11.53%), amikacin (*n* = 7, 6.73%), cefotaxime (*n* = 2, 1.92%), cefepime (*n* = 2, 1.92%), vancomycin (*n* = 1, 0.96%), meropenem (*n* = 1, 0.96%), and gentamicin (*n* = 1, 0.96%) (Table 4). Patients receiving two antibiotics of vancomycin and meropenem had abnormal ultrasound results. But, the administration of other antibiotics led to more normal sonographic findings. Pearson’s Chi-square analysis also showed that there were significant correlations between gender and antibiotic type (*p* = 0.0001) and hospitalization stay (*p* = 0.001). Thus, a proper choice of used antibiotics can significantly reduce fever degree and duration, and subsequently, the hospitalization stay of children with AP.

**Table 4:** The type of identified pathogens and used antibiotics to treat 104 children with AP

Pathogen type	Frequency (n [%])	Antibiotic therapy type	Frequency (n [%])
*Escherichia coli*	96 [92.31]	Ceftizoxime	78 [75.0]
Gram-negative *Bacillus*	2 [1.92]	Ceftriaxone	12 [11.53]
GroupB *Streptococcus*	2 [1.92]	Amikacin	7 [6.73]
*Acinetobacter*	1 [0.96]	Cefotaxime	2 [1.92]
*Enterococcus*	1 [0.96]	Cefepime	2 [1.92]
*Klebsiella*	1 [0.96]	Vancomycin	1 [0.96]
*Staphylococcus aureus*	1 [0.96]	Meropenem	1 [0.96]
		Gentamicin	1 [0.96]

Overall, ESR and CRP values had recorded for 91 and 99 patients with AP, respectively. The minimum, mean, and maximum levels of ESR and CRP were 3 mm/h and 1 mg/L, 46.5 mm/h and 58.7 mg/L, and 108 mm/h and 118 mg/L, respectively (Table 1). The elevated ESR (> 10 mm/h) and CRP (> 10 mg/L) levels [22] were observed in 84 (92.30%) and 82 (82.82%) patients, respectively. Therefore, only 7 and 17 patients respectively had an ESR and CRP within a normal range. Accordingly, high amounts of these hematological factors may be effective in the diagnosis of patients with AP. The minimum, mean, and maximum amounts of BUN and SCr were 5 and 0.05 mg/dL, 11.31 and 0.57 mg/dL, and 62 and 2.4 mg/dL, respectively. Also, the minimum and maximum eGFR amounts respectively were 17.2 and 134 mL/min/1.73 m^2^, while the average value of this index was calculated to be 68.94 mL/min/1.73 m^2^ (Table 1). Also, the lowest, average, and highest values of WBCs and Hb were 3.6/mm^3^and 7.6 ng/mL, 14.90/mm^3^and 11.02 ng/mL, and 34/mm^3^and 14.7 ng/mL, respectively (Table 1). The frequency of leukocytosis and anemia in the studied population was 81.73% (*n* = 85) and 36.53% (*n* = 38), respectively. The mean Na and K amounts in blood samples were 140.7 and 4.36 mEq/L, respectively. The lowest and highest values of Na and K were 131 and 3.5 mEq/L, and 152 and 5.8 mEq/L, respectively (Table 1).

In general, 67.3% of the patients had normal sonographic examinations. A good inter-observer agreement was found for ultrasound imaging in the study years (kappa value: 0.751–0.837). Sonographic findings showed the presence of hydronephrosis, cystitis, and renal anomalies in 22 (21.15%), 9 (8.65%), and 2 (1.92%) patients with AP, respectively (Table 3). However, none of the patients showed ureteral stones on their ultrasound images. Consequently, hydronephrosis was the most common abnormality detected in the ultrasonography of kidneys and urinary tract. Since sonography results of 70 patients (67.3%) were not in favor of pyelonephritis, ultrasound alone cannot be a valid diagnostic technique for this disease.

## Discussion

A particular interest in the field of pediatric studies is early diagnosis and discrimination of AP from other UTIs such as cystitis because of long-term morbidities and serious complications. Girls than boys are more susceptible to get involved in AP due to their shorter urethras [23]. However, boy populations with UTIs typically have underlying anatomical or functional abnormalities of the genitourinary tract with a higher primary scarring rate [23]. *E. coli* is the most common AP-causing pathogen in children. It was previously shown that this Gram-negative rod bacterium was present in 69% of the English patients with AP, while *K. pneumoniae*, *E. faecalis*, *Proteus mirabilis*, and *Pseudomonas aeruginosa* caused 3–6% of AP in other patients [24]. Mahmoudi et al. [25] and Sarvari et al. [26] earlier reported the frequent presence of *E. coli* in urine samples collected from Iranian pediatric populations with AP. Since *E. coli* is the main pathogen responsible for AP, the antimicrobial sensitivity profile of this member of the family Enterobacteriaceae should be considered as a principle in determining the empirical therapeutic protocols [17]. The number of symptomatic patients with AP in this study was higher compared to that of in the study of Mahmoudi et al. (12.8%) [25]. Although Muhammad et al. [27] explained that constipation is a frequent and overlooked problem in children with UTI symptoms, most patients in this study (79.81%) did not show constipation complications. Pelvic floor dynamics are significantly worsened with this complication. The existence of large stool masses accompanied by volitional holding delays/prevents the complete bladder emptying because of pain with defecation. This mechanism leads to the high accumulation of post-void residuals facilitating bacterial colonization (such as *E. coli*) in the bladder [28, 29]. The guideline available in the American Academy of Pediatrics recommends pyuria as a clinical factor to diagnose UTIs [30]. Pyuria was the common factor in most children with AP in the present study (88.46%). The pyuria percentage in this research was in agreement with the findings of Nickavar and Sadeghi-Bojd [31] and Shaikh et al. [32] who respectively reported 81 and 87% pyuria among Iranian and American children with AP. Also, Renata et al. [33] pointed out that the frequency of pyuria among the studied Israeli infants and children was 93.5%. They also showed that patients with pyuria significantly had higher concentrations of urinary interleukin-6 (UIL-6) and interleukin-8 (UIL-8) [33]. As a laboratory factor in diagnosing AP in children, we found that the presence of nitrite in urine samples was more pronounced than hematuria. The positive nitrite reaction is a specific test so that it only detects Gram-negative coliforms, whereas atypical pathogens (such as *Pseudomonas* and Gram-positive organisms) cannot be identified [34]. Moreover, high urine-specific gravity can remarkably reduce the sensitivity of this test [35]. However, as the presence of atypical pathogens in urine samples was insignificant, this factor in our study was relatively appropriate to detect children with AP. The prevalence rate of leukocytosis as a common feature of inflammatory reaction in the current study (81.73%) was much more than that of (56%) in Ayazi et al. [22]. Lee et al. [36] also mentioned leukocytosis as an important risk factor for renal scar formation in Korean infants with the first episode of AP. Shah and Upadhyay [37] earlier reported a significant increase in leukocytosis among children with AP. CRP is synthesized by the liver in response to inflammatory cytokines, particularly UIL-6. ESR shows the complete acute phase process mainly as a response to the production of fibrinogen [38]. The levels of CRP and ESR also were much more than the measured amounts in studies conducted by Ayazi et al. [22] and Naseri [39]. This fact showed that the high amounts of these hematological factors were risk factors to develop renal scars in the long-term follow-up. Jung and Lee [40] proved that UTI infants with a higher CRP significantly had higher cortical defect on an acute dimercaptosuccinic acid (DMSA) scan. Rodríguez et al. [41] revealed that CRP can be considered a valid test to diagnose febrile UTI with high sensitivity (83.3%) compared to UIL-6 (77.8%). In contrast, Lin et al. [42] reported that ESR and CRP had a relatively low sensitivity to diagnose UTI in febrile infants.

Ultrasound not only is a non-invasive, easily repeatable, safe, and relatively cheap technique to diagnose infectious diseases but also it does not require any sedation and is easy to examine bedside [43]. Other benefits of ultrasound are the lack of ionizing radiation hazards, general availability, and patient acceptability [44]. Sonography examinations showed that only 34 patients were in favor of AP with a more appearance of hydronephrosis. The size of kidneys during AP may be enlarged so that they have hypoechoic parenchyma with a loss of the normal corticomedullary junction [45]. Our ultrasonographic findings demonstrated that this imaging tool did not have sufficient adequacy to diagnose AP as it may miss parenchymal and perinephric abnormalities [46]. Thus, other diagnostic methods in combination with ultrasound should be used to monitor AP in pediatric patients due to the disadvantages such as the accepted low sensitivity, high operator dependence, and required expertise for the data interpretation [47, 48]. It has been recently reported that the combined use of ultrasound with DMSA scintigraphy [49], voiding cystourethrogram [50], and technetium-99 m DMSA (^99m^Tc-DMSA) scintigraphy [51, 52] can highly improve the diagnostic accuracy of AP among pediatric patients.

### Limitations

Although there was a normal range of patients in this study similar to other studies, more count of subjects in this six-year cross-sectional study could present the data with better reliability and generalizability. The evaluation of AP in larger population sizes from several hospitals in different geographical areas is recommended because this work was performed in a single academic hospital, limiting its generalizability to other centers and settings. Another limitation is no record of the used dose of each antibiotic to decide about the therapeutic potential and its effects on the fever duration and hospitalization stay. Hence, the present study cannot be a criterion to choose appropriate antibiotic treatments for AP in children populations. The other limitation was the failure to record inflammatory markers in medical files. The assessment of molecular mechanisms regulating the inflammatory profile could contribute to more comprehension of the overall pathogenesis and clinical outcomes of pediatric UTIs to define the best type and dose of antibiotics with the lowest acquired resistance.

## Conclusions

The present study showed that the AP in most Iranian children was symptomatic and mainly caused by *E. coli*. This bacterial infection was highly associated with some urine (pyuria and urine nitrite) and hematological (high levels of CRP and ESR, and leukocytosis) factors. Furthermore, the most common antibiotics used to treat AP were ceftriaxone and amikacin. Even though the management of AP is a challenging and controversial process in pediatric populations, the detection, treatment, and follow-up of children with AP should be conducted according to the efficient medical guidelines by pediatricians and renal specialists. Findings obtained from accurate markers for the early prognosis, diagnosis, and treatment of AP based on urine and hematological analyses, antibiotic therapies, and imaging tools would be helpful to overcome this common health problem in childhood. As ultrasonographic findings were not efficient to differentiate pediatrics with and without AP, the use of other imaging diagnostic tools alone or in combination with ultrasound can provide better diagnostic performance.

## Data Availability

All the data of this case series are available on request from the corresponding author. The data are not publicly available due to privacy or ethical restrictions.

## Abbreviations

AP: Acute pyelonephritis
BMI: Body mass index
BUN: Blood urea nitrogen
CRP: C-reactive protein
^99m^Tc-DMSA: Technetium-99 m dimercaptosuccinic acid
ESR: Erythrocyte sedimentation rate
Hb: Hemoglobin
HPF: High-power field
PUC/NUC: Positive/negative urine culture
RBCs: Red blood cells
SCr: Serum creatinine
UTI: Urinary tract infection
VUR: Vesicoureteral reflux
UIL: Urinary interleukin
WBCs: White blood cells
